# Component of sexual health services for vaginismus management: A qualitative study

**DOI:** 10.1371/journal.pone.0283732

**Published:** 2023-08-09

**Authors:** Mojdeh Banaei, Nourossadat Kariman, Vida Ghasemi, Nasibeh Roozbeh, Maryam Jahangirifar

**Affiliations:** 1 Mother and Child Welfare Research Center, Hormozgan University of Medical Sciences, Bandar Abbas, Iran; 2 Midwifery and Reproductive Health Research Center, School of Nursing and Midwifery, Shahid Beheshti University of Medical Sciences, Tehran, Iran; 3 Department of Public Health, Asadabad School of Medical Sciences, Asadabad, Iran; 4 School of Nursing and Midwifery, Faculty of Medicine, Nursing and Health Sciences, Monash University, Melbourne, Australia; King Khalid University, SAUDI ARABIA

## Abstract

**Background:**

Provision of sexual health services requires gender-sensitive management, facilities, and staff, as well as planning for gender-sensitive caregivers and education. Couples suffering from vaginismus face many types of barriers to accessing sexual health services. This qualitative study was conducted to explain the needs of sexual health services in women with primary vaginismus in Iran.

**Methods:**

This qualitative study was conducted through the participation of 20 participants including service providers, women with vaginismus and their husbands in 2022, Iran. The samples were selected using purposive sampling method and considering the maximum variation. For data collection, in-depth semi-structured individual interviews were conducted and continued until data saturation was reached. The collected data were analyzed in MAXQDA10 software using conventional content analysis approach based on the criteria proposed by Graneheim and Lundman.

**Results:**

Data analysis led to the emergence of three main themes: 1) Comprehensive preventive sex education which included the three categories of sex education in the education system, premarital sex education through the health system, and sex education through the media with scientific content; 2) Efficient sexual health clinics which included three categories of therapist’s skills, empowerment of sexual therapist, and structural features of sexual health clinics and cultural considerations in establishing sexual health clinics; and 3) Protocol for management and treatment of sexual problems which consisted of sexual education and counseling content, treatment requirements, and sex education approaches.

**Conclusion:**

Based on the results of the study, comprehensive preventive sex education through the education system and the Ministry of Health can improve the attitudes of adolescents and young people. Moreover, it can take a fundamental step in solving sexual problems by providing the infrastructure necessary for the establishment of efficient sexual health clinics and protocols required to manage and treat such problems.

## Introduction

Sexual health is an essential aspect of personal health that can affect anyone at any age or stage of life [1]. Regardless of their disability or illness, all people have sexual feelings and have the right to enjoy their sexual activities. Accordingly, these people need to have an appropriate awareness of their sexual and fertility issues in order to achieve a satisfactory and desirable life [2]. The provision of sexual health services requires gender-sensitive management, facilities, and staff, as well as planning for gender-sensitive caregivers and education [3]. More precisely, sexual health education should be gender sensitive, age-appropriate, and proportional to ethnicity, religion, values, and cultural norms [4]. Sex education programs are required for both those who have started and those who have not started their sexual activity such as children, adolescents and young people [5]. The promotion of sexual and fertility information in men and women can improve their quality of life and general health status [2].

Based on the evidence, inadequate sexual knowledge has been identified as the most prevalent cause of sexual problems [6]. Insufficient sex education can lead to the development of vaginismus as a sexual disorder [7]. Vaginismus is a sexual dysfunction that prevents sexual penetration via involuntary and frequent spasm of the muscles in the one-third of the vagina’s outer part [1]. Based on previous studies conducted on women admitted to sexual health clinics, the prevalence of this disorder has been 7.8% in Italy [8] and 6.4% in Portugal [9]. In a study in Iran, about 33% of Iranian women expressed their experience of pain or fear during intercourse [10].

Treatment management of women with vaginismus is multifaceted and its various dimensions should be considered. To correct any misinformation about sexual function and genitals in women with vaginismus, a step-by-step training approach and the introduction of exercises to achieve therapeutic goals are recommended [11]. Vaginismus treatment exercises should be easy, enjoyable, and compatible with the patient’s lifestyle [12]. Given the importance of the family in Iranian society and the role of couples’ sexual health in the stability and positive functioning of the family, sexual health education should be integrated into premarital education and couple empowerment [13] which can effectively prevent vaginismus. However, it was shown in a study that the content of sex education offered in universities and the education provided at the time of marriage cannot qualitatively meet the need for sexual awareness [7]. Some studies demonstrated that taboo of sexual issues, lack of sexual information, stigma and sexual discrimination are the challenges of sexual health in Iran [14].

Therefore, improving the quality of sex education in premarital counselling classes is another appropriate strategy which can diminish the problems of sex education in the prevention of vaginismus by providing more transparent education in this regard and also continuing education for couples after marriage. The components of reproductive and sexual health services in Iran include family planning, preconception care, pregnancy care, postpartum care, middle and old age care, pre-marriage training classes, counseling and treatment of sexual diseases and puberty counseling that often done by midwives [15]. Given that sex-related issues are supposed to be taboo in many Asian countries, and considering insufficient sexual health education [16], the incidence of vaginismus can be directly related to these issues. Couples with vaginismus, consequently, may encounter numerous barriers to accessing sexual health services. This qualitative study was conducted to explain the needs of women with primary vaginismus to sexual health services in Iran.

## Materials and methods

### Design

Using conventional content analysis approach based on the criteria proposed by Graneheim and Lundman this qualitative study was conducted [17]. We used consolidated criteria for reporting qualitative research (COREQ) in our study. This checklist consist 32 items in 3 scopes including: (i) research team and reflexivity, (ii) study design and (iii) data analysis and reporting [18].

### Sample/Participants

This study was conducted from March to July 2022, to explain the needs of women with primary vaginismus to sexual health services in Iran. The research setting in this study included sexual health clinics and selected offices in Iran, Tehran and even places such as the workplace, which were preferred by the participants. Inclusion criteria were: married Iranian women aged 18–50 years with primary vaginismus, whose vaginismus was decidedly diagnosed based on a sex therapist examination and DSM-IV diagnostic criteria, without other acute and chronic physical or mental diseases and key informant including service providers (reproductive health, psychology, Psychiatrist, sexology and midwifery specialists) with at least 2 years of work experience; and spouses of these women with at least 6 months of marital experience and absence of any sexual dysfunction. The participant’ willingness to withdraw from the study was the only exclusion criteria.

### Data collection

For data collection and conducting interview, the first author (MB) referred to sexual health clinics and selected offices in Tehran, Iran. In order to access a rich source of information related to the research question, key informants, women with vaginismus and their husbands who were rich source of information were participants in this study. Key informants were people who either based on their job and education or based on experience, had useful information about the research. Purposive sampling method was used through considering maximum variation in terms of age, education level, employment status, and marriage duration. Women with vaginismus and their spouses by face-to-face method and key informants by telephone and email were invited to participate in the study. Before conducting the interviews, the researcher explained the objectives of the research, the confidentiality of information, the voluntary participation to the participants and obtained their written and oral informed consent for participating in the research and recording their voices. All interviews were conducted by the first author, who holds a Ph.D. in Reproductive Health and has passed qualitative research courses. The whole process of data collection and analysis was performed under the supervision of a professor specializing in qualitative research who was also faculty members of medical universities. In-depth semi-structured individual interview using open-ended questions in Persian was used as the data collection method. All interviews were conducted in a private setting and even places such as the workplace, which were preferred by the participants, and the participants were nicknamed. If voice recording was not allowed by any participant, note taking was used instead.

The data collection tool was the guide to semi-structured questions. The interview began with open-ended questions about the sexual health service needs of women with vaginismus, such as "What is the situation of sexual health services for women with vaginismus? Explain it". "What do you think about the sexual health service needs of women with vaginismus?" "What strategies do you suggest to improve these services?" During the interview, whenever the researcher felt the need to have a deeper perception of the issue and more clarification, she used probe questions such as "How?", "What do you mean?", and "Please explain more if you can". Each interview lasted for 45–60 minutes, and all interviews were recorded. In addition to interviews, field notes and memos were also used for data collection. Generally, we invited 22 participants for an interview, 1 key informant did not accept the invitation to participate in the interview due to lack of time and a woman with vaginismus due to her unwillingness to participate in the interview. Although data saturation was reached with 17 interviews, three additional interviews were conducted for more certainty. Finally, 20 in-depth individual interviews were conducted with seven women with primary vaginismus, two spouses of the women with primary vaginismus as well as eleven service providers including 2 reproductive health specialists, 2 psychologists, a psychiatrist, a social medicine specialist with sexual medicine fellowship and psychosexology, 3 sexologists and 2 midwifes (Table 1).

**Table 1:** The Socio-demographic of participants (women with primary vaginismus and key informants) (N = 20).

Row			Age (Year)	Education	Occupation	Work experience/ Married duration (year)
1	Women with primary vaginismus		40	A.D. *	Housewife	12
2			31	A.D.	Employee	6
3			35	B.Sc. **	Teacher	7
4			29	B.Sc.	Housewife	3
5			24	B.Sc.	Housewife	2
6			31	M.Sc.***	Teacher	9
7			39	Ph.D.****	Employee	15
8	key informants	Spouse	30	B.Sc.	self-employment	3
9			35	B.Sc.	Employee	6
10		Experts	41	Ph.D. of reproductive health/ Sexual Medicine Fellowship	Faculty member	13
11			38	Ph.D. of reproductive health	Faculty member	8
12			53	Social medicine specialist with sexual medicine fellowship and psychosexology	Physician	17
13			36	M.Sc. of midwifery	Midwife	11
14			29	M.Sc. of midwifery	Midwife	6
15			39	Ph.D. in Clinical Psychology	Faculty member	12
16			51	Ph.D. in Clinical Psychology	Faculty member	29
17			30	Psychiatrist	Physician	12
18			50	Ph.D. in Sexual Health	Sexologist	8
19			41	Ph.D. in Sexual Health	Sexologist	9
20			47	Ph.D. in Sexual Health	Sexologist	11

^*^ A.D.: Associate’s degree; **B.Sc.: Bachelor’s degree; **** M.Sc.: Master’s Degree; ****Ph.D.: Doctor of Philosophy

### Ethical considerations

This study was approved by the Ethics Committee of Hormozgan University of Medical Sciences (Ref. ID: IR.HUMS.REC.1401.122). Confidentiality of the collected data was ensured, and written informed consent was obtained from all research participants. Also, participants were free to decline participation or withdraw at any stage of the research process. All interviews were recorded with the permission of the participants, and all the audio files were securely stored in password-protected computers.

### Rigour

Guba and Lincoln criteria were used to evaluate the rigor and trustworthiness of the data [19]. The credibility of the research was confirmed through allocating sufficient time to data collection, investigator triangulation, expert check-peer check, the researcher’s prolonged engagement with the data, and review of the participants (member checking). In this regard, interview texts along with the extracted codes were given to two participants to ensure that the findings were consistent with their experiences. To increase the dependability the research team used an external auditor to allow for investigate all the steps such as data collection and data analysis. External auditors were an unbiased person in research and specialist in qualitative research. In addition, other research members reviewed the interview text and coded interviews and any disagreement was discussed a resolved by discussion among research members. For triangulation, different sources (women with vaginismus, spouses and specialists) were used. In addition to interviews, field notes and memos were also used for data collection.

For transferability and comprehensiveness, the samples were selected considering the maximum variation. To ensure confirmability of the data, the researcher abandoned all her assumptions and thoughts and carefully documented all the research steps, including data collection, data analysis, and code formation.

### Data analysis

Data were analyzed in MAXQUDA10 concurrently with data collection using conventional content analysis approach based on the criteria proposed by Graneheim and Lundman [20]. After transcribing the recorded interviews, they were carefully reviewed by the first author (M.B), -as a Ph.D. of reproductive health who had passed qualitative research courses- several times to achieve an accurate understanding of the interview contents. The whole process of data analysis was supervised by N.K. as expert in qualitative research and faculty members of Shahid Beheshti University of Medical Sciences. In addition, other research members reviewed the coded interviews and any disagreement was discussed a resolved by discussion among research members. This method promotes reflexivity by examining an individuals’ own construction of knowledge [21], effectively improves overall transparency and trustworthiness while also ensuring the methodological soundness and practical relevance of the research [22]. All stages of the research were recorded very precisely for the possibility of an audit. All elements of trustworthiness, including credibility, dependability, and transferability were considered in this study. The researcher abandoned all her assumptions and thoughts and quotations from the participants were used in presenting the results.

After reviewing the text of interview, the text was divided into meaning units. Meaning units were condensed while preserving the meaning and labelled with codes. Similar codes were then categorized into subcategories, and the subcategories were classified into a category based on common properties. The latent content of the similar categories was eventually formulated as a theme. In this process we used an external auditor to allow for investigate the process of data analysis. In addition, if was any disagreement, research team discussed and resolved it.

## Results

Twenty participants, including seven women with primary vaginismus, two spouses of the women with primary vaginismus and eleven specialists participated in this study. Demographic information of the participants is given in Table 1. Data analysis led to the emergence of three main themes: "Comprehensive preventive sex education", "Efficient sexual health clinics" and "Protocol for management and treatment of sexual problems". Themes, categories, and subcategories are shown in Table 2.

**Table 2:** Theme, categories and subcategories of components of sexual health services for vaginismus management.

Theme	Category	Subcategory
**Comprehensive preventive sex education**	Sex education in the country’s education system	Sex education in schools and universities
		Methods and content of sex education in schools and universities
		Correcting sexual attitudes of adolescents by sex education
	Sex education through the media with scientific content	Sex education through movies and TV programs
		Sex education through social networks
	Premarital sex education through the health system	Premarital sex education content
		The necessity of premarital sex education
**Efficient sexual health clinics**	Therapist’s skills	Communication skills
		No judgment in the counseling process
		The ability of creating a sense of comfort in the couples during the treatment process
		Sufficient experience and knowledge about sexual problems
	Empowerment of sex therapists	Increasing the awareness and skills of medical staff to provide sexual services
		Adding the course of sexual dysfunction to the educational curriculum
	Structural features of sexual health clinics	Increased access to sexual counseling centers
		The need for providing information about sexual counseling centers
		The leveling of sexual health services
		The need for covering sexual services by insurance
		The provision of sexual services by multidisciplinary teams
	Cultural considerations in establishing sexual health clinics	Culture-building with regard to sexual counseling centers
		Attention to gender in providing sexual services
**Protocol for management and treatment of sexual problems**	Sexual education and counseling content	Increasing couples’ awareness of sexual issues, training of physical
		Mental exercises for pelvic floor muscle relaxation
		Educations on sexual skills
		Education for promoting one’s self-confidence and eliminating sense of guilt
		Trainings for improving one’s sexual potency (self-efficacy)
		Correcting couples’ beliefs and attitudes
	Treatment requirements	Need to consider the treatment based on individual requirements
		The structure of treatment sessions
		The need for continuous sexual exercises
		The need for follow-up and support in the treatment process by the therapist
		The companionship of the spouse in the treatment process
		The need for the couple’s desire and commitment to solve the problem
	Approaches to sex education	Using people with similar experiences in the process of treatment
		Education through new educational models

### 1. Comprehensive preventive sex education

Comprehensive preventive sex education refers to sex education in the country’s education system, premarital sex education through the health system, and sex education through the media with scientific content. In the written program of comprehensive preventive sex education, teenagers and young people form their attitudes, opinions and values about sexual behavior in a correct way by acquiring correct and necessary information and knowledge about sexual issues.

#### 1.1. Sex education in the country’s education system

This category reflects the significance of sex education in the country’s education system. The participants of this research pointed to sex education in schools and universities through appropriate educational methods and content, and so that the sexual attitudes of teenagers and young people can be improved through sex education. With regard to this category, a participant stated:

*“It would be better to start such teachings from high school in the form of a course and a 50-page booklet; like sports courses*, *an hour should be allocated to sex education each week*. *From high school*, *these issues can be taught to both boys and girls*: *teachings about the anatomy of their bodies and the opposite sex; sexually transmitted diseases; ways to prevent and treat them*, *etc*.*”* (P.4)

#### 1.2. Sex education through the media with scientific content

In this category, the participants provided suggestions on sex education through videos and TV programs as well as through social networks. With regard to using social media for sex education, a medical specialist said:

*“Generally speaking*, *access to authentic sources of sexual health is one of the rights to sexual health*. *Firstly*, *we don’t have Persian sites in this regard*, *and secondly*, *if someone is fluent in English*, *they cannot use such sites properly*. *To my mind*, *it would be better if a team of experts collect*, *translate and culturally adapt such sexual material and put it on reputable websites so that others can use it”* (P.12)

#### 1.3. Premarital sex education through the health system

Based on the experiences of the participants, this category consisted of two subcategories: premarital sex education content and the necessity of premarital sex education. In fact, for the participations, it is better to get comprehensive information into pre -marriage educational content in order to improve the quality of marital life and greater satisfaction with the sexual life of the couple. The need for premarital sex education was clarified by one of the participants as follows:

*“They encourage us to marry soon; how can we get married when we don’t have enough information*?! *Nowadays*, *marriage is so complicated; couples should be educated about these sexual issues before marriage”* (P.2)

The content of premarital sex education provided by the health system was another point made by the participants:

*“It would be fantastic if premarital classes used videos and photos to explain women’s sexual problems such as vaginismus; for example*, *couples should be informed that some types of hymens don’t bleed during the penetration*. *I think we should teach men how to arouse women sexually*, *how to have successful intercourse*, *and how to choose a proper position*. *Preventive methods should also be taught to them*.*”* (P.3)

### 2. Efficient sexual health clinics

Efficient sexual health clinics refers to therapist skills, sexual therapist empowerment, structural features of sexual health clinics, and cultural considerations in establishing sexual health clinics. To create sexual health clinics, its standards need to be met to achieve more effective and quality consequences. These clinics can provide a platform for sex education and increased sexual health literacy.

#### 2.1. Therapist’s skills

The experiences of the study participants with regard to this category gave shape to four subcategories: communication skills; no judgement in the counselling process; the ability to create a sense of comfort in the couples during the treatment process; and sufficient experience and knowledge about sexual problems. With regard to this category, two of the participants said:

*“It is so important that a midwife*, *doctor*, *psychologist*, *or any other specialist*, *who is supposed to work in the field of sexual health*, *be able to establish a good relationship with their clients; they also have to educate couples through using understandable words which are proportional to the culture of our society*.*”* (P.6)

*“I believe that service providers should have adequate knowledge; only specialists who are knowledgeable in that area can help”* (P.1)

### 2.2. Empowerment of sex therapists

This category consisted of two subcategories: increasing the awareness and skills of medical staff to provide sexual services and adding the course on sexual dysfunction to the educational curriculum. In fact the knowledge, skill, and experience of the therapist play a decisive role in solving the sexual problems of the clients. A number of key informants in the present study stated in this regard:

*“It is so important that a midwife*, *doctor*, *psychologist*, *counselor*, *or any other person who is working in the field of sexual health be so educated and skillful*. *So then*, *they at least don’t cause any more harm to the client*. *There are numerous weaknesses in this system according to which some believe that we don’t have sex therapists and our service providers can’t treat sexual dysfunctions; so*, *if they can’t*, *the system should empower them to acquire the necessary skills”* (P.16)

#### 2.3. Structural features of sexual health clinics

The suggestions of participants in this category included: increased access to sexual counseling centers; the need for providing information about sexual counselling centers; levelling of sexual health services; the need for covering sexual services by insurance; and the provision of sexual services by multidisciplinary teams. With regard to this category, a number of the key informants stated:

*“In my opinion*, *the best thing to do is to integrate sexual health services into the first level of health services*, *or PHC*. *Part of people’s problems are solved with a series of simple information and awareness provided by the service providers; and wherever they couldn’t solve these problems*, *the clients will be referred to higher levels”* (P.15)

*“Sexual problems need to be defined for the reproductive health and obstetrics groups that deal mostly with women; the services provided to them should also be covered by insurance so that the patients can attend all the counselling sessions for free*. *Otherwise*, *they will not attend the sessions because of their high costs”* (P.11)

#### 2.4. Cultural considerations in establishing sexual health clinics

A number of participants referred to cultural considerations and they stated that about sexual health clinics, there should be culture building among the people so that they can use these centers properly. Also, sexual services in these centers should be gender sensitive so that all people can refer for services regardless of their gender. a key informants made the following comment with reference to this category:

*“People will find solutions to their sexual problems if they are informed by the media or any other institution; however*, *if they are not informed*, *they will be confused where they should refer*. *So*, *it is really important to inform them about such centers”* (P.10)

### 3. Protocol for management and treatment of sexual problems

Protocol for management and treatment of sexual problems refers to sexual education and counseling content, treatment requirements, and approaches to sex education.

#### 3.1. Sexual education and counseling content

Contrary to the existence of many cultural, religious, social and political sensitivities regarding sexual affairs in society, which makes teaching such subjects difficult and sometimes impossible, it can be done by designing appropriate and targeted educational counseling programs that match people’s needs to achieve acceptable results in this field. This category includes the subcategories of increasing couples’ awareness of sexual issues, training of physical and mental exercises for pelvic floor muscle relaxation, education on sexual skills, education for promoting one’s self-confidence and eliminating sense of guilt, training for improving one’s sexual potency (self-efficacy), and correcting couples’ beliefs and attitudes. Some key informants noted the following with relation to this category:

*“In their treatment sessions*, *we must talk to them about the anatomy and physiology of their body and sexual problems so that they can gain a better understanding of*, *for example*, *vaginismus and why it happens; doing so*, *they know that they should not blame themselves and that the situation is completely involuntary*.*”* (P.13)

*“One of the best and most effective treatments for these people is regular desensitization in the form of pelvic floor exercises by inserting objects (e*.*g*., *dilators) that they should gradually begin to insert small to large objects into their vagina*.*”* (P.20)

*“These women usually suffer from a sense of inferiority and low levels of self-esteem; they have a feeling of inability and we should remind them of their values as women in the process of treatment in order to restore their self-confidence*.*”* (P.14)

### 3.2. Treatment requirements

Participants pointed out that the treatment process of different couples is different and that it is better to be based on individual treatment requirements. It is best to determine the structure of treatment sessions in the first session and tell the clients. Also, in the treatment process, the need for continuous sexual exercises, the companionship of the spouse in the treatment process, the need for the couple’s desire and commitment to solve the problem. It is necessary to follow-up and support in the treatment process by the therapist. With regard to this category, some key informants mentioned the following:

*“Well*! *It differs for various people*, *and we can’t write a single structure for everyone*. *The most important story we have to listen to in the first sessions of the treatment process is the history of the patient’s vaginismus*. *Vaginismus treatment requires at least two or three hours of history*.*”* (P.18)

*“It is really significant to convince them that they should do their exercises regularly”* (P.19)

*“In couples who support each other*, *the treatment process goes much better*. *That is due to the supportive role of the spouse*.*”* (P.12)

*“In my opinion*, *it is really important that they be enthusiastic and attend the treatment sessions regularly and be committed to their treatment*.*”* (P.11)

### 3.3. Approaches to sex education

Based on the experiences of the participants, this category consisted of two subcategories: using people with similar experiences in the process of treatment and education through new educational models. In the present study, using the experiences of people with similar experiences in the treatment process was actually a kind of education through peers, which aimed to share the experiences of people with vaginismus and use them in the education process. With regard to this category, a key informant mentioned:

*“Sometimes we can convey a series of information to these people through peer education; because sometimes people help each other using this method as a positive empowering factor*. *This means that when these experiences are read by another woman in the form of a story*, *it will be useful and lead to the belief that she is not the only person with this problem”* (P.17)

## Discussion

This qualitative study was conducted to explain the sexual health service needs of women with primary vaginismus in Iran. Data analysis led to the emergence of three main themes; "comprehensive preventive sex education", "efficient sexual health clinics" and "protocol for management and treatment of sexual problems". Comprehensive preventive sex education consisted of the three categories of sex education in the education system, premarital sex education through the health system, and sex education through the media with scientific content.

With regard to the category of sex education in the education system, the participants provided suggestions about sex education in schools and universities, methods and content of sex education, and improving the sexual attitudes of adolescents through sex education. Many misconceptions about sexual issues are formed in childhood and adolescence, which can affect one’s sexual function in adulthood [23]. In line with the results of the present study, the participants in the study of Bostani Khalesi et al. (2020) considered sex education through universities as one of the strategies for empowering young people in the field of sexual health [24]. Therefore, it is recommended that sex education be provided in the form of written and age-specific educational programs with observance of educational-moral principles and customary norms.

Regarding sex education through the media with scientific content, some participants provided suggestions on sex education through videos and TV programs as well as through social networks. Sex education through radio and television is one of the most important strategies for empowering people with regard to sexual health [24]. It was argued in another study that insufficient credible sex education resources will make people turn to satellite products and Internet pornography to meet their sexual education needs [25]. Since the media play a crucially effective role in the field of sexual health in creating a culture and breaking irrational taboos, It is essential to implement programmes with the purpose of promoting sexual health and providing proper information for the improvement of sexual health [26].

With regard to premarital sex education and the need for premarital sex education, the category of premarital sex education through the health system was extracted from the experiences of some participants. Formal premarital sexual health education was one of the methods for the empowerment of couples in the area of sexual education [27]. In line with the results of the present study, Pourmarzi et al. (2013) showed that in order to empower couples who are about to get married, comprehensive sexual health information needs to be included in premarital educational content to provide better quality and healthier married lives [28]. Formal premarital sexual health education was one of the methods for the empowerment of couples in the area of sexual education [29].

The theme of efficient sexual health clinics included three categories of therapist’s skills, empowerment of the sexual therapist, and structural features of sexual health clinics and cultural considerations in establishing sexual health clinics. Norallahi et al. (2018) acknowledged that sexual health therapists should be cautious, not judge, not label clients, and respect the patient’s rights [30]. Moreover, healthcare providers need to be able to communicate with these patients and diagnose their problems [31]. Therefore, basic communication skills such as listening without judgement are an integral part of providing support for women in the treatment process [32].

In the present study, empowerment of the sex therapist was referred to by some participants. Service providers who are usually involved in vaginismus assessment rarely have sufficient expertise and skill to diagnose muscle spasms [33, 34]. In a study, 92% of the participants believed that midwives could be the best counsellors for sex education, sexual skills, and sexual health [35]. In sexual instructions, the therapist should provide the clients with the necessary information, thereby removing the ambiguities and creating the correct sexual attitude and behavior [36]. Accordingly, the knowledge, awareness, and experience of the therapist play a decisive role in solving the sexual problems of the clients [37].

A number of participants referred to the structural features of sexual health clinics. Bostani Khalesi et al. (2020) maintained that cooperation should be established among the sectors of health, education, the Ministry of Culture and Islamic Guidance, and the Ministry of Sport and Youth with regard to sex education [24]. The lack of specialized counselling and clinical systems for resolving sexual and marital problems was also noted by the participants of another study. In fact, this issue was one of the weaknesses of the country’s healthcare system, which should be solved through training sexual health specialists [38]. Thus, free sexual health services should be provided, or the cost of services should be determined according to the announced tariff by the competent authorities, and these services should be available to clients at the right time and place. Moreover, clinic staff should be provided with initial training and annual retraining [30]. Also, sexual health services should be an integral part of the reproductive health services system [39]. Cultural considerations were also considered by the participants, in this study. Bostani Khalesi et al. (2020) emphasized the universality of sex education and culture-based sex education. Gender-sensitive education was another issue mentioned by the participants. In other words, considering the age and gender of the target group or learners was considered to be an integral and influential factor in empowerment-based education [24].

The protocol for management and treatment of sexual problems consisted of three categories: sex education and counseling content; treatment requirements; and sex education approaches. In most cases, the cognitive perception of a vaginismus patient regarding the physical appearance and capacity of their vagina is distorted, and correcting the cognition and misconceptions of such patients can effectively lead to their treatment [40]. Thus, conventional treatment of vaginismus mainly includes sexual education and counseling, relaxation exercises, and the use of different-sized dilators or other alternative tools, all of which are included in cognitive behavioral therapy (CBT) [41]. With regard to sexual knowledge, it has been shown in various studies that sexual skills training can cause positive feelings, intimacy between the spouses, forgiveness, increased marital relationships and, consequently, marital conflict resolution and marital satisfaction [42].

Also in this study, a number of cases were mentioned as treatment requirements. In line with the present study, Norallahi et al. (2018) pointed out the need for informed consent and confidentiality in the process of treating sexual problems [30]. One of the factors discovered in the present study was the need to consider the treatment based on individual requirements through obtaining an accurate history of the couple. Thus, the first session is the most important and sometimes the only treatment session [43]. Since sexual intercourse is a mutual behavior, sexual problems will be more successfully treated when a mutual identity is given to them, that is, when each of the spouses does not blame themselves or the other [44]. As such, the cooperation of the spouses is one of the pillars of giving a mutual identity to these problems [45]. In line with the findings of our study, another study emphasized the continuous follow-up and availability of the therapist via social networks. Doing so, their probable problems during the exercises would be solved immediately [46]. Therefore, it can be concluded that in order to treat sexual problems, specific attention should be paid to some medical requirements.

A number of key informants of the present study referred to the category of approaches to sex education. In the present study, using the experiences of people with similar experiences in the treatment process was actually a kind of education through peers, which aimed to share the experiences of people with vaginismus and use them in the education process. Peer education was considered in a study as one of the empowerment-based strategies for sexual health education [24]. Moreover, using the experiences of people with shared experiences in the treatment process is actually a kind of group therapy. Group therapy provides a strong support system for a patient who feels ashamed, anxious, or guilty about a particular sexual problem [47].

Based on the results of the present study and other similar studies, vaginismus can be introduced as a multidimensional phenomenon. Therefore, by providing the necessary infrastructure, such as the recognition of sex education programs for all members of society, holding sex education courses for children, and adolescents to empower parents in the education of children and streamlining premarital education classes, vaginismus can be prevented to some extent. As such, it is recommended that the Ministry of Health, Treatment, and Medical Education and other related organizations provide health, counselling and treatment services, establish, equip, and expand specialized centers in this area, and create a referral system to these centers in the general body of health services. Additionally, the lack of knowledge and information about sexual issues at the level of healthcare personnel shows the need for education and training of these personnel in the form of retraining courses. Thus, considering the significance of sexual health and its role in strengthening the family foundation and promoting the mental health of individuals, especially young couples, it is recommended that these courses be held.

### Limitation and strengths of the study

The strengths of the present study included interviewing key informants (spouses and experts) in addition to women with vaginismus. The study limitations included the selection of samples from only married women with vaginismus given the sociocultural conditions in Iran, which prohibits single, divorced and widowed females from having sex.

## Conclusion

Based on the results of the present study, comprehensive preventive sex education through the education system and the Ministry of Health can improve the attitudes of adolescents and young people and prevent sexual problems such as vaginismus to a large extent. Moreover, by providing the necessary infrastructure for the establishment of efficient sexual health clinics and the protocol for the management and treatment of sexual problems, a fundamental step was taken to solve this sexual dysfunction. Therefore, it is recommended that the Ministry of Health, Treatment, and Medical Education and other related organizations provide health, counselling and treatment services, establish, equip, and expand specialized centers in this area; and create a referral system to these centers in the general body of health services.
